# Analytical Performance of COVID-19 Detection Methods (RT-PCR): Scientific and Societal Concerns †

**DOI:** 10.3390/life11070660

**Published:** 2021-07-06

**Authors:** Roberto Verna, Walter Alallon, Masami Murakami, Catherine P. M. Hayward, Abdel Halim Harrath, Saleh H. Alwasel, Nairo M. Sumita, Ozkan Alatas, Valeria Fedeli, Praveen Sharma, Andrea Fuso, Daniela Maria Capuano, Maria Capalbo, Antonio Angeloni, Mariano Bizzarri

**Affiliations:** 1 In Unam Sapientiam, 00185 Rome, Italy; roberto.verna@fondazione.uniroma1.it; 2 WASPaLM, CT Corporation, P.O. Box 4349, Carol Stream, IL 60197-4349, USA; danicapuano@libero.it; 3 Academy for Health and Clinical Research, 00185 Rome, Italy; 4 Department of Clinical Laboratory, Hospital de Clínicas, Facultad de Medicina, Universidad de la República, Montevideo 11000, Uruguay; alallon@adinet.com.uy; 5 Department of Clinical Laboratory Medicine, Gunma University Graduate School of Medicine, 3-39-15 Showa-Machi, Maebashi 371-8511, Japan; mmurakam@gunma-u.ac.jp; 6 Health Science Center, Departments of Pathology and Molecular Medicine, and Medicine, Room 2N29A, McMaster University, 1200 Main Street West, Hamilton, ON L8N 3Z5, Canada; haywrdc@mcmaster.ca; 7 Department of Zoology, College of Science, King Saud University, Riyadh 11451, Saudi Arabia; halim.harrath@gmail.com (A.H.H.); salwasel@ksu.edu.sa (S.H.A.); 8 Grupo Fleury, Central Laboratory Division, Hospital das Clínicas, Faculdade de Medicina, Universidade de São Paulo, São Paulo 05508, Brazil; nairo.sumita@grupofleury.com.br; 9 Department of Medical Biochemistry, Eskisehir Osmangazi University Medical School, Eskisehir 33400, Turkey; oalatas@ogu.edu.tr; 10 Department of Experimental Medicine, Sapienza University, 00160 Rome, Italy; valeria.fedeli@uniroma1.it (V.F.); andrea.fuso@uniroma1.it (A.F.); antonio.angeloni@uniroma1.it (A.A.); 11 Department of Biochemistry, All India Institute of Medical Sciences, Jodhpur 342005, India; sharmapr@aiimsjodhpur.edu.in; 12 Azienda Ospedaliera Ospedali Riuniti Marche Nord (DG), 61121 Pesaro, Italy; maria.capalbo@ospedalimarchenord.it

**Keywords:** COVID-19, SARS-CoV-2, RT-PCR, false positive, false negative, pandemic, clinical pathology

## Abstract

*Background.* Health and social management of the SARS-CoV-2 epidemic, responsible for the COVID-19 disease, requires both screening tools and diagnostic procedures. Reliable screening tests aim at identifying (truely) infectious individuals that can spread the viral infection and therefore are essential for tracing and harnessing the epidemic diffusion. Instead, diagnostic tests should supplement clinical and radiological findings, thus helping in establishing the diagnosis. Several analytical assays, mostly using RT-PCR-based technologies, have become commercially available for healthcare workers and clinical laboratories. However, such tests showed some critical limitations, given that a relevant number of both false-positive and false-negative cases have been so far reported. Moreover, those analytical techniques demonstrated to be significantly influenced by pre-analytical biases, while the sensitivity showed a dramatic time dependency. *Aim.* Herein, we critically investigate limits and perspectives of currently available RT-PCR techniques, especially when referring to the required performances in providing reliable epidemiological and clinical information. *Key Concepts.* Current data cast doubt on the use of RT-PCR swabs as a screening procedure for tracing the evolution of the current SARS-COV-2 pandemic. Indeed, the huge number of both false-positive and false-negative results deprives the trustworthiness of decision making based on those data. Therefore, we should refine current available analytical tests to quickly identify individuals able to really transmit the virus, with the aim to control and prevent large outbreaks.

## 1. The Challenge: Scientific and Political Problems

The spread of the SARS-CoV-2 pandemic around the globe has created several critical problems that should be addressed concomitantly, with some problems requiring higher prioritization. The pathophysiology of the disease (that affects presentation and complications), early detection of infectious individuals, and the need to provide adequate treatment are among the topics that require very timely and appropriate management. With any pandemic, recognizing the etiological factor (the virus) is an essential starting point. A key second step is to establish a reliable and specific method to detect viral infection (especially the RNA messenger or viral antigens) and/or the related immunological consequences (specific antibodies, belonging to the IgG, IgM, and IgA clusters) in suspected cases. Undoubtedly, the COVID-19 epidemic has promoted the repositioning of laboratory medicine to a more visible and strategic place in healthcare systems to deal with the current disease and to prepare for and prevent future pandemics [1]. 

Awareness of the need for timely development of testing capabilities has led several regulatory agencies—including FDA—to find acceptable levels of compromise between test reliability and quick turnaround by readily available and useful diagnostic tools. This has shortened timelines for assessing mandatory requirements considerably, in some cases, to just a few weeks. This approach to making rapid approval decisions has led to the approval of several diagnostic tools without long-term information on reliability, performance, and diagnostic accuracy, which affect test validity [2].

## 2. Diagnosis of SARS-CoV-2 Infection

The current diagnostic procedures for the identification of SARS-CoV-2-infected individuals are primarily based on two distinct approaches [3]. The first is the direct detection of the virus or its components. This can be carried out by a culture of the virus, detection of one or more of its proteins or other components, or via amplification of viral nucleic acids through RT-PCR techniques (usually known as “molecular tests”). The last two are the most frequently used techniques during the present pandemic, and the main aim of the present paper is to address some critical considerations regarding the molecular tests. 

The second approach is the use of immunological tests to detect the host’s immune response triggered by the virus through the detection of specific antibodies (IgM, IgG, but also IgA). Some specialized laboratories are also able to test the host’s cellular immune response to the virus. In addition, whole-genome sequencing has been applied to determine the sequence of the SARS-CoV-2 virus in a sample, with the goal to identify changes in sequence, or so-called variants [4] to further understand the epidemiology and viral factors that predict disease transmission and severity. However, this is clearly an extremely expensive approach that cannot be pursued in the frameshift of a rapid, population-based, screening.

The three most used immunological assays are enzyme-linked immunosorbent assays (ELISAs), chemiluminescence assays (CLIAs), and lateral flow assays (LFAs) [5]. Virus neutralization tests are an additional type of specialized immunoassay to specifically detect neutralizing antibodies, but this type of method is primarily used for assay validation and research purposes. Preliminary reports on ELISA assays to detect viral infection have indicated a fairly good correlation between antibody titers and virus-neutralizing antibodies [6]. In addition to the assays described, many other assays have been developed, as summarized elsewhere [7].

## 3. Preanalytical Issues Affecting the Diagnosis of COVID-19

It is widely acknowledged that pre-analytical factors represent a major source of error in laboratory testing [8]. Such errors include the inappropriate collection of biological material; inadequate sample storage/transportation (including the presence of additive or cellular components that may interfere with the assay, in some cases, due to whole-blood freezing); pipetting errors; contamination; or sample mismatch. Sample contamination is a key issue to address to ensure the quality of reverse transcription–polymerase chain reaction (RT-PCR) assays, given that even trace amounts of foreign nucleic acids can jeopardize test findings. A second critical source of pre-analytic error is the use of improper procedures for the collection of nasopharyngeal specimens. Indeed, the recommended procedure is not so straightforward: a nasopharyngeal sample should be obtained by inserting the swab far into the nostril parallel to the palate to reach the deepest target area, maintaining the swab in place for few seconds for the absorption of secretions, then immediately putting the swab into a sterile tube [9]. Failure to comply with the recommended practices can cause several diagnostic errors [10].

## 4. Limits of Current RT-PCR Tests

According to several reports, the diagnostic accuracy of many of the currently available RT-PCR tests for SARS-CoV-2 may be lower than optimal, as false-positive, and false-negative results are seen in a small but significant proportion of individuals. These shortcomings have been ascribed to several factors, including lack of harmonization of primers and probes; technical and analytical errors; and the absence of validation by independent third parties [11]. The US Food and Drug Administration Emergency Use Authorization (EUA) and Instructions for Use (IFU) documents outline the currently approved virology tests for SARS-CoV-2, which are largely unstandardized [12]. For example, FDA data on EUA SARS-CoV-2 virology tests show a wide range of limits of detection (LoD), spanning >5 orders of log_10_ differences across different assays. Undoubtedly, assays with higher LoDs will likely miss more infected patients. These metrics are of critical importance because each 10-fold increase in the LoD of a COVID-19 viral diagnostic test is expected to increase the false-negative rate by 13% [13]. Beyond “this variable performance” reported in IFUs for EUA tests, key attributes of many tests, such as primer sequences, protocol steps, or viral gene targets, are either unclear or missing [12]. 

**Figure 1:**
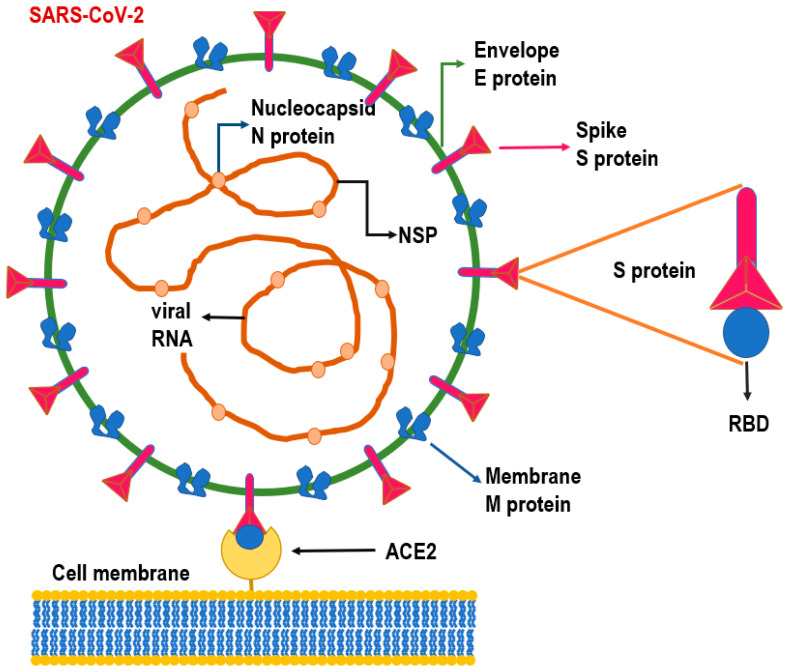
Structure of SARS-CoV-2 virus. RNA of the viral genome is constituted by two genes—ORF1a and ORF1b—encoding for the nonstructural proteins NSP, while small genomic region includes S (spike protein), M (membrane protein), E (envelope protein), and N (nucleocapsid protein) genes. The S protein interacts with the angiotensin-converting enzyme 2 (ACE2) receptors of the host cell membrane through a receptor-binding domain (RBD) positioned on the spike protein. Once the ACE2/RBD interaction has been established, the virus can be endocytosed into the cell.

Several different primer-probe sets for use in SARS-CoV-2 detection assays have been developed and are currently in use. Overall, these tests show high specificity when tested against a panel of samples positive for several other respiratory viruses, while their sensitivity is highly variable [14]. It is noteworthy that assays that use the CDC N2 primer–probe, developed by the Division of Viral Diseases at the Centers for Disease Control and Prevention (CDC) [15]—and the Corman E-gene primer–probe [16], display high sensitivity, even in presence of low genomic equivalents of viral RNA. According to the Corman protocol (further endorsed by World Health Organization (WHO)) [17], the *E*-gene assay is used as a first-line screening tool, which is then followed by a confirmatory assay, based on an RNA-dependent RNA polymerase gene or an *N* gene test, respectively. On the contrary, the test supported by the CDC involves three *N* gene primer/probe sets, and as such, it is designed for detecting SARS-like coronaviruses (one primer/probe set), as well the specific detection of SARS-CoV-2 (two primer–probe sets) (Figure 1). The panel may eventually include a primer/probe for recognizing the human RNase *P* gene (*RP*) in control samples and clinical specimens. 

Differences in primers concentrations and/or in DNA probe lengths used by different protocols have raised some concern, given that it has been argued that those settings do not conform with the FDA protocols, and this may be one of the factors that lead to false-positive results [18]. Furthermore, a nearly 100-fold difference in LoD has been recorded among different assays that use different genes and probes, which cannot be considered equivalent [19]. 

Usually, when RT-PCR analyses are designed for the first time, amplification is followed by a second step where a separation technique (i.e., electrophoresis) or a sequencing run is used to confirm that the amplified sequence was correct. Of course, this cannot be routinely performed in population-based screening, but more accurate characterization of the PCR reactions used to reveal SARS-CoV2 infection is advisable.

Furthermore, a nearly 100-fold difference in LoD has been recorded among the different assays that use different genes and probes; therefore, these tests cannot be considered equivalent [19]. Usually, RT-PCR analyses are followed by a second step where a separation technique (i.e., mobility of the amplified fragment on a gel) is used to confirm that the amplified substance was correct. Unfortunately, in screening settings for ascertaining SARS-CoV-2 positivity, RT-PCR is generally not followed by a second, confirmatory step. When in diagnostic settings, with a high pretest probability of SARS-CoV-2, RT-PCR assays may provide important confirmation of the suspected diagnosis, based on clinical and radiological data. However, when used as a tool to perform screening surveys, extensive RT-PCR testing cannot suffice in providing reliable results. For other endemic viral diseases—including HCV, HIV, and chronic HBV—positive results for specific viral tests occur after suggestive symptoms appear, and RT-PCR tests are usually preceded by serological analyses that greatly reduce the risk of false-positive results from molecular tests.

It is embarrassing to the field that very few studies have addressed the prevalence of false-negative results for SAR-CoV-2 molecular assays. Yet, the critical validation of diagnostic tests given emergency approval by regulatory agencies cannot be indefinitely postponed [20]. For SARS-CoV-2, it is unclear if the sensitivity of any authorized commercial test has been assessed in the proper way. Current rules permit indirect demonstration of the test performance by applying the RT-PCR test to known positive material from symptomatic people or contrived specimens: such assessments likely overestimate test sensitivity as swabs may lack viral particle [21]. A reference standard for determining the sensitivity of SARS-CoV-2 tests in asymptomatic people is still lacking. At the very beginning of the epidemic in China, at least two studies reported on the impact of missing truly infected patients due to overestimation of RT-PCR tests sensitivity, resulting in a false-negative rate ranging from 2 to 30% [22,23]. A recent meta-analysis carried out on 12,057 COVID-19 confirmed cases reports that there is substantial and largely unexplained heterogeneity in the proportion of false-negative RT-PCR results for SARS-CoV-2, which cannot be explained by differences in the statistical approach used in different studies [24]. Differences in reliability rates might result from differences in the type of specimen collected, the time to onset of symptoms, the surmised viral load, at the time when the test is performed, to product-specific factors related to the RT-PCR kit used. All this information is either partially or unreported in most studies. Noticeably, one report [25] demonstrated that up to 54% of COVID-19 patients may have, during the first phase of their illness, an initial false-negative RT-PCR. Accordingly, the field is faced with very low certainty of evidence. Additionally, some published studies have several biases (protocol violation to rule in/rule out the presence of SARS-CoV-2) and/or fail to report key index test characteristics.

*Site of collection of the biological specimen*. The analytical specificity of an analytical test is its capability to recognize solely the molecule that is to be identified—both qualitatively and quantitatively—in presence of interfering substances or antigenically overlapping compounds. For molecular tests, the relevant abundance of RNA is essential for bestowing sensitivity to the assay. In turn, RNA abundance is strongly dependent on the type and site of collection of the biological sample. Indeed, the sensitivity of RT-PCR for SARS-CoV-2 is higher for lower respiratory samples than for upper respiratory tract samples [25,26]. Surprisingly, as reported in a preliminary study of 213 COVID-19 cases, and 866 respiratory samples obtained onset from different sites of collection during the first week after symptom, the highest positive rate (>80%) was recorded in salivary samples, followed by nasopharyngeal (72–73%) and throat swabs (60–61%) [27,28].

A systematic review, performed on pooled data for 8136 clinical specimens tested for SARS-CoV-2 by RT-PCR, substantially confirmed results for different types of specimens: [29] specimens from the lower respiratory tract showed a positive rate of 71.3%; bronchoalveolar lavage fluid and saliva samples had the highest positivity rates of 91.8% and 87.8%, respectively; oropharyngeal swab, feces, and blood showed the lowest positivity rates (7.6%, 32.8%, and 1%, respectively), whereas no SARS-CoV-2 was detected in urine samples. Another study showed that the rate of RT-PCR detection of SARS-CoV-2 in patients is as high as 93% in bronchoalveolar lavage fluid, 72% in sputum, and 63% in nasopharyngeal swabs, while it is only 32% in pharyngeal swabs and 29% in stool [28]. Yet, till now, there has not been an independent assessment of the overall trustworthiness of different RT-PCR tests, a global comparative analysis of these tests applied to different settings, or consensus on the most reliable samples across different tests (nasopharyngeal versus saliva). A few studies found that saliva samples yielded greater detection sensitivity and more consistent findings throughout the course of infection when compared with samples taken via nasopharyngeal swabs [30,31]. The very preliminary investigations, using saliva specimens, displayed huge variability in sensitivity estimates, ranging from 70 to 100%, when compared to the throat and nasopharyngeal swabs tested by RT-PCR [32,33]. As for analyses carried out on nasopharyngeal samples, differences in reported sensitivities may reflect differences in the timing of sampling, relative to the onset of infection. Indeed, the highest sensitivity was observed among hospitalized and critically ill patients [34], while the lowest sensitivity has been recorded when saliva samples were collected during the late phase from symptom onset or exposure [35]. A recent meta-analysis suggests that the diagnostic accuracy of saliva-based nucleic acid amplification testing is similar to that of nasopharyngeal swabs, especially in the ambulatory setting [36]. Overall, the current information suggests that saliva, when obtained in the early phases of COVID-19 infection (10 days after the symptom onset), is reliable, and it may be more practical to collect saliva than nasopharyngeal swabs for the screening and diagnosing of the virus [37].

*Viral variants*. An important issue of concern that has been increasing during recent months is the increasing recognition of different viral haplotypes, due to the occurrence of recombination and/or mutation [38]. High variability in genomic pattern and nucleotide sequences is in keeping with the rapid spread of the SARS-CoV-2 pandemic [39]. Furthermore, as observed by an extensive survey carried out on 220 SARS-CoV-2 genomic sequences, different strains of SARS-CoV-2 might coexist [40], thus conferring further complexity to the diagnostic assessment of the infection. Moreover, another study revealed that ~79% of primer-binding sequences in at least one gene used in RT-PCR were mutated, compared to the original SARS-CoV-2 genome (Wuhan-Hu-1, NC_045512) [41]. A bewildering viral diversity has been found even in single patients, with a median number of four/five intra-individual variants [38,42]. It should be stressed that such heterogeneity in viral RNAs not only could help explaining differences in intra-individual immunological response and clinical pathophenotype [43] but can also undermine the accuracy of RT-PCR detection [44]. Consequently, it is currently assumed that about 10–15% of SARS-CoV-2 variants were not detectable by at least one of the commercialized primers [45].

## 5. Time Dependency and False-Negative Results

The kinetics of viral load is another factor that varies between individuals and is influenced by the patients’ epidemiological history, immune response, and treatment or medication effects [46]. It is another factor that can contribute to false-negative results. 

A false-negative case of SARS-CoV-2 infection is defined as an individual with suspected infection and an initial negative result, as determined by the RT-PCR test, with a positive result on a subsequent test. False-negative results among hospitalized patients have received considerable attention at the beginning of the COVID-19 pandemic, especially in those patients in which clinical and radiographic findings were at odds with negative test results. Most of these false-negative data can be explained by considering the evolution over time of the viral load. Viral RNA is usually undetectable during the first two weeks after the infection (when the patient is generally asymptomatic) as well as two-four weeks later after the onset of clinical symptoms. Viral RNA load declines quickly two and three weeks after the appearance of symptoms. This indicates that there is a narrow window for detecting viruses, and there is a potential for false-negative results if tests are performed outside the period of detectable viral load [47]. 

In a literature review and pooled analysis, Kucirka et al. [48] analyzed the rate of false-negative RT-PCR performed on nasopharyngeal swabs of symptomatic patients, with respect to the timing of symptom onset. The probability of a false-negative result decreased from 100% on day 1 to 67% (CI, 27–94%) on day 4. On the day with onset of symptoms the probability of a false-negative rate was 38% and then decreased to 20% 3 days after symptom onset), while, during the asymptomatic stage (1–4 days), the false-negative rate ranged from 100 to 94%.

**Figure 2:**
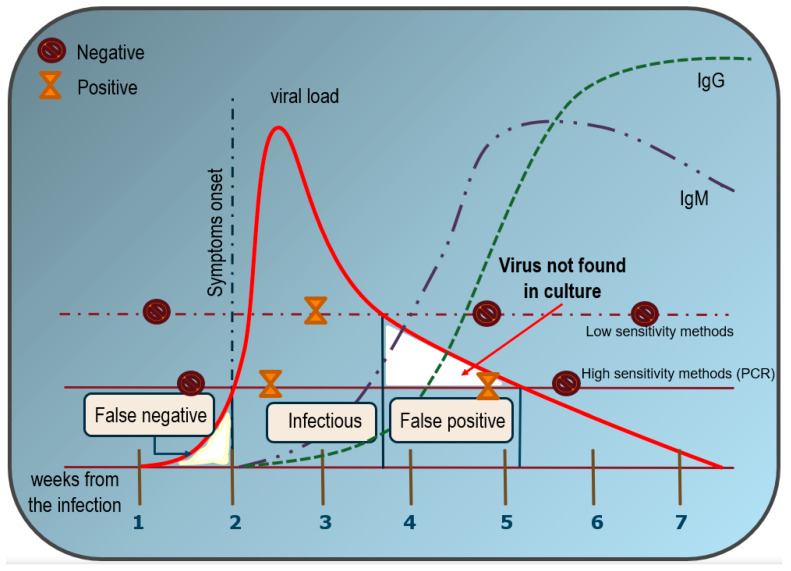
Estimated over time likelihood of SARS-CoV-2 virus detection with RT-PCR tests and serological assays in respect to symptom onset. The figure has been reconstructed on the basis of several reports and the estimated values are only approximate averages. Viral load shows an asymmetric distribution, with an extended tail. The maximum probability to detect infectious individuals happens after the first week since the infection and lasts for almost three weeks. Since then, it is quite unlikely to identify subjects that could truly transmit the disease. As a consequence, no virus development occurs in culture from biological samples in which RT-PCR positivity has been ascertained when the cycle threshold is very high (a Ct < 30 is suggested, but it may vary depending on the swab procedure accuracy). Unlike low-sensitivity methods (dashed line), the high sensitivity of the RT-PCR method could therefore lead to disclosing more positives that, on the other hand, are not contagious (solid line). Conversely, false-negative results may occur during the first week, when active viral replication does not reach the sensitivity level required by the RT-PCR test. For comparison, serological analysis shows a steady increase in both IgM and IgG values only after 4–5 weeks. Thereafter, IgM progressively decreases after 7–8 weeks, whereas IgG still persists for several months.

Overall, the sensitivity of RT-PCR testing is therefore severely limited. The sensitivity in identifying SARS-CoV-2 bearing individuals by RT-PCR test ranges from 44% to 80% [49], as being significantly influenced by viral shedding and by the time of sample collection when compared to the onset of the infection, as already observed for other coronaviruses [50]. As a result, it is probable that large numbers of mild, asymptomatic, or pre-symptomatic COVID-19 cases are not detected by current testing efforts [51] (Figure 2).

## 6. Critical Objectives and False-Positive Results

In the public health setting, when we are faced with a highly contagious epidemic, a critical objective is to optimize the early identification of infected individuals that can (potentially) transmit the microbe. This is especially relevant in detecting SARS-CoV-2-positive people, who, in many cases, are unaware of their infection. Epidemiologic data have shown that a huge reservoir of asymptomatic/paucisymptomatic subjects has driven the diffusion of the current pandemic of COVID-19 [52]. Clinical and epidemiological studies show that the infectious period begins about 2 days after exposure and continues to about 12 days after symptom emergence [53]. Overall, the infectious period lasts, on average, for about two weeks, and during this time, the viral load (RNA copies per mL) is likely to increase progressively. Therefore, timely identification of persons that can actually transmit the virus is mandatory to put in place confinement measures and identify probable contacts. 

However, positive individuals do not overlap automatically with those that can effectively transmit the virus, although some variants are emerging to have higher rates of infectivity. Accordingly, the ideal test is not necessarily the one that determines whether a person has any evidence of SARS-CoV-2—as determined by current PCR-RT-based swabs—but the one that quickly and accurately identifies those that are truly capable of transmitting the infection in order to prevent large outbreaks. In other words, we need a reliable test that could identify the “infecting” individuals. The canonical rules, established by Koch’s postulates [54], stating that the microorganism must be isolated from the infected individual and should show growth in culture, have been confirmed in animal models of SARS-CoV-2 [55]. Indirect evidence of virus replication capability is provided by tests that identify viral subgenomic mRNA, that can be transcribed in infected cells or detected in infected cells by fluorescent in situ hybridization techniques that recognize the virus associated with cells [56]. Of course, this kind of testing is neither rapid nor cheap, and it is therefore not applicable, so far, for large population screening. However, even if the antigenic and the genetic “rapid” testing still remain the most useful for tracing the spread of the virus, it is desirable that more diagnostic tests are settled up in view of a second phase of the “SARS-CoV2 affair” when the urge for rapid population screening will be less mandatory and more accurate diagnosis will be relevant.

Overall, all authorized molecular tests (that amplify viral RNA) have higher sensitivity than antigen tests. Yet, new rapid diagnostic tools, based on lateral flow assays for COVID-19 antigens, promise to be a reliable alternative for identifying infectious patients [57]. Such tests capture viral proteins in a lateral flow format that can give results in few minutes and thus greatly help to rapidly identify those individuals at the highest risk for transmitting disease [58]. Although less sensitive than reverse transcriptase–polymerase chain reaction tests, early data suggest that antigen tests can be used to diagnose individuals that are infectious with COVID-19 [59]. In this study, positivity for viral antigens matched with virus isolation using an optimal SARS-CoV-2 isolation procedure, i.e., antigen detection strictly correlates with the presence of virus and can therefore represent a reliable tool in monitoring the evolution of viral infectivity within the population (epidemiological surveillance). 

*Data misinterpretation.* Diagnostics can be used in various manners, the so-called use cases: triage of symptomatic individuals in an epidemic or endemic setting, triage of at-risk presymptomatic and symptomatic individuals in endemic settings, and confirmatory testing. The use determines the way in which diagnostic tests are optimally used, namely, the critical issue is to correctly distinguish between diagnostic and surveillance (screening) tests, the latter being conceived for ascertaining the epidemiological course of the epidemic, whereas the former is more critical for the management of individual patients.

For epidemiological testing, the key question is not how well molecules can be detected in a single sample but how effectively infections can be detected in a population by the repeated use of a given test as part of an overall testing strategy—the sensitivity of the testing regimen. Clinical tests are designed for assessing the reliability of diagnosis in symptomatic individuals and require high sensitivity. On the other hand, tests used in surveillance regimens should be tailored to detect those individuals that can really diffuse the virus (true “infecting” persons). RT-PCR detects RNA, not an infectious virus; thus, its ability to determine the duration of infectivity of patients is very limited, while infectivity is a critical determinant in informing public health guidelines/interventions. As already observed in other instances, a critical drawback of RT-PCR tests (but that can be extended to serological tests too) is that they cannot determine virus infectivity: overall, RT-PCR sensitivity is good but its specificity for detecting the replicative virus is poor [60]. 

A recent paper has highlighted that infectivity—recognized by the capability to retrieve the virus growing in culture according to Koch’s postulate—is dramatically impaired when cycle threshold (Ct) values are >24 [61]. A direct relationship has been observed between Ct values of RT-PCR and the viral load, even if the relationship is less than linear for low viral loads [62]. Noteworthy, for every one-unit increase in Ct, the odds ratio for infectivity decreased by 32%. Another report shows that for Ct = 25, the virus can be identified in up to 70% of samples, while at Ct = 30, this value drops to 20%; for Ct = 35, only in 3% of cultures the virus can retrieved [63]. Therefore, the straightforward consequence is that almost 50% of newly diagnosed “positive” patients are in fact noninfectious individuals, given that in these cases, the PCR Ct averages >30, indicating low or even no viral counts [64]. Indeed, besides the fact that these low levels of detectable RNAs could be explained by an early or late-stage infection, the prolonged duration of the RNA-positive tail—during which individuals continue to “release” viral RNA or its fragments—indicates that a relevant proportion of infected persons are recognized once the infectious period has already passed [65]. It is generally agreed that replication-competent virus cannot be successfully cultured more than 9 days after the onset of illness, with a statistically estimated likelihood of recovering replication-competent virus that approaches zero by 10 days [66,67]. Conclusively, “after about 8 days of onset of symptoms, infectious virus is no longer present in respiratory tract specimens, despite high amounts of SARS-CoV-2 RNA that are measurable by RT-PCR” [68].

Overall, it has been estimated, in a different cohort of patients, that 88% and 95% of their specimens no longer yielded replication-competent virus after 10 and 15 days, respectively, following symptom onset [69,70]. This means the current COVID-19 test standards for the PCR test would pick up an excessive number of *false positives*. However, despite these warnings, an update to the CDC instructions for PCR testing from 1 December 2020, still uses a Ct of 40 [71].

Nonetheless, the implications are relevant, as hundreds of thousands of people presently being confined based on positive RT-PCR tests are likely to have already passed the transmissible phase of their infection. These results were anticipated by several investigations [56,72] and prompted different scholars to propose a cutoff Ct value at Ct ≤ 30, with a duration of eviction of approximately 10 days [73]. Interestingly, prolonged (i.e., with a duration > 20 days) persistence of positive RT-PCR test, with low Ct values, may indicate subgroups of infected patients with immunocompromised state or more severe disease, as suggested by van Kampen [74]. Additionally, it may be surmised that tests with high Ct thresholds may detect not just live viruses but genetic fragments, leftovers from other infections (i.e., other Coronavirus for which a cross-reactivity has been documented) [75,76] that pose no particular risk. Again, these considerations—albeit correct—hardly overcome the wall of the “field practice.” As a matter of fact, to be able at ascertaining a Ct cutoff to discern between infecting and noninfective individuals, one should be sure the starting specimen are homogeneous. Actually, the pre-analytic bias plays a non-neglectable role in the execution of the swab since different operators introduce a great variability in the sampling. It is clearly arguable that two different specimens taken from the same individuals can result in very different Cts depending on how accurately the swab has been executed.

*The New York Times* recently raised the alarm that the number of amplification cycles needed to find the virus “is never included in the results sent to doctors and coronavirus patients, although it could tell them how infectious the patients are. In three sets of testing data that include cycle thresholds, compiled by officials in Massachusetts, New York, and Nevada, up to 90 percent of people testing positive carried barely any virus” [77].

It is therefore mandatory to use tests that could capture infected people while they are still infectious. Identification of such persons is a *critical objective* deemed to reduce the population prevalence of a respiratory virus. In this respect, as stressed by a recent paper, “the benchmark standard clinical polymerase chain reaction (PCR) test fails when used in a surveillance regimen” [67]. Indeed, the distribution over time of RT-PCR positivity in infected people shows that the diagnostic test still remains positive for longer times after the stage at which an individual is no longer infectious [78].

Unfortunately, regulatory agencies have not established distinct and clear rules for validating, as separate tests, those to be used as analytical diagnostic tools versus tests for ascertaining infectivity and hence public health efforts to reduce community transmission. Moreover, a reliable assessment of the course of the epidemic worldwide needs different countries to adopt a common approach for validating performances of both diagnostic tests and tests used to screen for infectious persons. This is especially urgent when epidemiological data (i.e., the number of true infectious people) serve as a base for political decision making. Conversely, differences in RT-PCR tests, LoDs, or Ct cutoff may contribute to explaining controversial epidemiological findings, given that some differences among countries with similar structural sociological traits (population mean age and density, quality of health services, geographical position) could be ascribed to variances in RT-PCR-based data acquisition. 

As already proposed [79], to improve its reliability, the RT-PCR test should be continuously calibrated against a reference culture in Vero E6 cells in which cytopathic effect has been observed. The cycle threshold values on each platform for patients who have positive and negative viral cultures should be correlated with RT-PCR results and the resulting calibration curve could then be performed to estimate virus viability from the cycle threshold with some certainty [80]. Moreover, evidence shows that viral loads are subject to a different evolution in symptomatic and asymptomatic people [81]. Conclusively, the generation of false-positive or false-negative test results jeopardizes the health of the individual patient and may also derange and disrupt the efficacy of public health policies, emergency plans, and restrictive measures established by national and international authorities for containing the outbreak.

Secondly, an alternative to RT-PCR tests could be represented by rapid lateral-flow antigen tests, and rapid lateral-flow tests based on CRISPR gene-editing technology [82]. These tools provide a sensitive and robust means for high-throughput COVID-19 diagnosis suitable for use in screening settings and display the potential to enable reliable large-scale diagnosis [83]. Notably, lateral-flow antigen tests do not have an amplification step, and their analytic limit of detection is usually 100/1000 times less than RT-PCR tests; to reliably identify the people that can really “transmit” the virus, such tests need to be adequately sensitive and would benefit from a clear cutoff value that is established to distinguish the “infectious” individuals among “positive” individuals. Undoubtedly, the adoption of such a strategy will have huge consequences for harnessing the epidemic outbreak and successfully manage the disease spreading. 

Altogether, these considerations further outline that clinical symptoms and molecular analytical tools should be conjugated to perform a correct diagnostic assessment. Some diseases can be diagnosed based on a test alone; most diseases, however, are defined by the cluster of symptoms and signs, in addition to test results. In no case can a medical diagnosis be replaced by simple molecular tests. Indeed, interpreting the results of RT-PCR requires consideration of patient characteristics, such as symptoms and their severity, contacts history, presence of preexisting morbidities and drug history, the cycle threshold value, the number of days from symptom onset to test, and the specimen donor’s age [84]. As markedly recommended by a recent *Cochrane* report, a single symptom or sign could not accurately diagnose COVID-19 [85]. Furthermore, the Oxford Centre for Evidence-Based Medicine suggests that “the PCR test positivity counts should include a standardized threshold level of detection, and at a minimum, the recording of the presence or absence of symptoms. As a disease, the COVID-19 case definition should constitute a disorder that produces a specific set of symptoms and signs. The in-hospital case definition should, therefore, also record the CT lung findings and associated blood tests” [86].

## 7. Conclusions

Reliability of analytical tools in identifying infected individuals, i.e., persons that can transmit the virus, is mandatory to assess the evolution of the SARS-Cov-2 pandemic and to provide solid foundations for policy decision making. The life and the liberty of hundreds of millions of people, as well as the economy of countries around the globe, ultimately depend on such appropriate assessments. Unfortunately, several scientific studies and administrative official reports indicate that epidemiological data related to the prevalence of COVID-19 infection among people are likely to be significantly biased by the relevant incidence of both negative- and false-positive results. As a matter of fact, optimization of testing capability, extensive clinical and epidemiological validation, and proper approval from regulatory agencies are still needed. 

To sum up, the following observations can be made:

(1) False-negative results may be ascribed to erroneous/flawed analytical performances as well pre-analytical issues, including inappropriate choice of timing of testing (i.e., pick-up of the biological sample could have been obtained at the wrong timing, too early or too late with respect to the onset of the infection).

(2) False-positive results may instead arise chiefly as a consequence of extended Ct, far beyond the recommended threshold of 30–33. Other causes of false-positive results include wrong timing, cross-reactivity among SARS-CoV-2, and other Coronaviruses. 

(3) Last, but not least, false-positive results should be ascribed to administrative errors and malpractices, as demonstrated recently in Italy. A survey reported by Il *Corriere della Sera* [87] shows that positive patients, albeit symptomless, have been persistently added to the pool of positive even though they may have fully recovered from the time of the initial test, taken several weeks before. 

Even in the ideal case in which a test displays both high sensitivity and specificity, the number of false-negative and false-positive would still compromise efficient control of the epidemic. It has been calculated that, with sensitivity and specificity in the range of 98% and 99%, respectively—a goal that currently is far from being achieved—a total of 1000 cases will be missed when 5 million people are tested, and another 49,500 individuals will receive false-positive results [88]. Recent data demonstrated that the true picture is even worse, with a much greater proportion of false-positive ascertained in many countries, including Italy and the USA. Consequently, many people may be unnecessarily quarantined, and the public health system unnecessarily burdened with managing contact tracing, confinement, and other measures. Therefore, it should be mandatory to “interpret” RT-PCR by jointly examining clinical and radiological data, in the context of the pretest probability of disease. For COVID-19, the pretest probability assessment includes symptoms, previous medical history of COVID-19, presence of antibodies, any potential exposure to COVID-19, and the likelihood of an alternative diagnosis [89], namely, when low pretest probability exists, positive results should be interpreted with caution and a second specimen tested for confirmation given that low levels of viral RNA (identified by RT-PCT tests that require high Ct values) do not equate with infectivity unless the presence of virus particles has been documented, as clearly recommended by the European Centre for Disease Prevention and Control [90].

These conclusions cast on doubt the use of RT-PCR swabs as a *screening procedure* for tracing the evolution of the current SARS-COV-2 pandemic. The huge number of both false-positive and false-negative results deprives the trustworthiness of decision making, which is also influenced by political factors. Health management during such a pandemic is severely compromised when tests for the infection provide unreliable results [91,92].

Therefore, we should search for a test that can quickly and reliably identify those individuals—including symptomless people—that are truly infectious and represent a potential vehicle of virus diffusion. Reliable assessment of such a parameter is indeed what we need to control and prevent large outbreaks, especially when we are using a more fitted epidemiological index to track the epidemic advancement [93]. Unfortunately, current widespread use of RT-PCR swabs appear to be a waste of resources, while the public health threat warrants large-scale testing—“it would be more effective to authorize a small number of well-designed, well-developed, and validated tests run on common high-throughput platforms, followed by a few point-of-care tests, all of which are manufactured in large quantities, than to simultaneously develop and authorize scores of diagnostics” [91]. Our review has broader consequences on how screening procedures should be performed. Specifically, it is urgent to set a threshold value for RT-PCR analysis, given that for Ct > 30–35 the incidence of false-positive results would account for the majority of positive tests. Furthermore, testing in the absence of other proven prevention strategies is unable to prevent outbreaks [94]. Health authorities should be asked to urgently address such issues in order to establish a proper strategy for managing the current pandemic, as well as prepare for future threats of a similar kind.

Given that such a problem has been addressed only marginally by few studies, an extended recognition aimed at assessing the incidence of both false-positive and false-negative results is urgently warranted. The present review attempts to provide a compelling survey of available published data, some studies may have been lost notwithstanding. However, some questions have been left aside and deserve to be studied by future inquiries. For instance, specific epidemiological investigations should be planned to evaluate how false negative/positive results arise in correlation with different levels of infection prevalence. In addition, it must be investigated the relationship between the disappearance of the virus (analyzed by culturing biological samples to find replicating viral particles) and the concomitant values of RT-PCR: how long RT-PCR stands positive since the virus has been removed from biological fluids? Moreover, this information needs to be complemented with data of seroprevalence, obtained by testing people about the presence of immunological markers (IgG, IgM, and IgA) that are the only trustworthy parameters that can provide a proper estimate of the virus diffusion into the general population. Even more important, appraisal of immunological status would allow appreciating the extent of the acquired immunization, a critical health indicator that, after more than 18 months of the pandemic, still needs to be acquired. Future studies should timely investigate those challenging questions to plan a better-tailored health strategy aimed at grasping the overall complexity of the current pandemic, as highlighted by the current review.

## Data Availability

Data were obtained from an extended survey based on published article reported.

## References

[1] Jacobs J., Hardy L., Semret M., Lunguya O., Phe T., Affolabi D., Yansouni C., Vandenberg O. (2019). Diagnostic Bacteriology in District Hospitals in Sub-Saharan Africa: At the Forefront of the Containment of Antimicrobial Resistance. Front. Med. (Lausanne).

[2] Lagu T., Kulkarni N., Mahant S., Shah S.S. (2021). The Light at the End of the Tunnel: Reflections on 2020 and Hopes for 2021. J. Hosp. Med..

[3] Vandenberg O., Martiny D., Rochas O., van Belkum A., Kozlakidis Z. (2020). Considerations for diagnostic COVID-19 tests. Nat. Rev. Microbiol..

[4] Gorbalenya A.E., Baker S.C., Baric R.S., de Groot R.J., Drosten C., Gulyaeva A.A., Haagmans B.L., Lauber C., Leontovich A.M., Neuman B.W. (2020). The species Severe acute respiratory syndrome-related coronavirus: Classifying 2019-nCoV and naming it SARS-CoV-2. Nat. Microbiol..

[5] Yan Y., Chang L., Wang L. (2020). Laboratory testing of SARS-CoV, MERS-CoV, and SARS-CoV-2 (2019-nCoV): Current status, challenges, and countermeasures. Rev. Med. Virol..

[6] Padoan A., Bonfante F., Pagliari M., Bortolami A., Negrini D., Zuin S., Bozzato D., Cosma C., Sciacovelli L., Plebani M. (2020). Analytical and clinical performances of five immunoassays for the detection of SARS-CoV-2 antibodies in comparison with neutralization activity. EBioMedicine.

[7] Sheridan C. (2020). Coronavirus and the race to distribute reliable diagnostics. Nat. Biotechnol..

[8] Lippi G., Simundic A.M., Plebani M. (2020). Potential preanalytical and analytical vulnerabilities in the laboratory diagnosis of coronavirus disease 2019 (COVID-19). Clin. Chem. Lab. Med..

[9] Interim Guidelines for Collecting, Handling, and Testing Clinical Specimens for COVID-19. https://www.cdc.gov/coronavirus/2019-ncov/lab/guidelines-clinical-specimens.html.

[10] Irving S.A., Vandermause M.F., Shay D.K., Belongia E.A. (2012). Comparison of nasal and nasopharyngeal swabs for influenza detection in adults. Clin. Med. Res..

[11] Loeffelholz M.J., Tang Y.W. (2020). Laboratory diagnosis of emerging human coronavirus infections—The state of the art. Emerg. Microbes Infect..

[12] MacKay M.J., Hooker A.C., Afshinnekoo E., Salit M., Kelly J., Feldstein J.V., Haft N., Schenkel D., Nambi S., Cai Y. (2020). The COVID-19 XPRIZE and the need for scalable, fast, and widespread testing. Nat. Biotechnol..

[13] Arnaout R., Lee R.A., Lee G.R., Callahan C., Yen C.F., Smith K.P., Arora R., Kirby J.E. (2020). SARS-CoV2 Testing: The Limit of Detection Matters. bioRxiv.

[14] Nalla A.K., Casto A.M., Huang M.W., Perchetti G.A., Sampoleo R., Shrestha L., Wei Y., Zhu H., Jerome K.R., Greninger A.L. (2020). Comparative Performance of SARS-CoV-2 Detection Assays Using Seven Different Primer-Probe Sets and One Assay Kit. J. Clin. Microbiol..

[15] Division of Viral Diseases (2020). 2019-Novel Coronavirus (2019-nCoV) Real-Time rRT-PCR Panel Primers and Probes.

[16] Corman V.M., Landt O., Kaiser M., Molenkamp R., Meijer A., Chu D.K., Bleicker T., Brünink S., Schneider J., Schmidt M.L. (2020). Detection of 2019 novel coronavirus (2019-nCoV) by real-time RT-PCR. Euro Surveill..

[17] Laboratory Testing for Coronavirus Disease 2019 (COVID-19) in Suspected Human Cases. Interim Guidance 2 March 2020. https://apps.who.int/iris/rest/bitstreams/1271387/retrieve.

[18] Lee S.H. (2020). Testing for SARS-CoV-2 in cellular components by routine nested RT-PCR followed by DNA sequencing. Int. J. Geriatr. Rehabil..

[19] FDA SARS-CoV-2 Reference Panel Comparative Data. https://www.fda.gov/medical-devices/coronavirus-covid-19-and-medical-devices/sars-cov-2-reference-panel-comparative-data.

[20] Woloshin S., Patel N., Kesselheim A.S. (2020). False Negative Tests for SARS-CoV-2 Infection - Challenges and Implications. N. Engl. J. Med..

[21] U.S. Food and Drug Administration Emergency Use Authorization (EUA) Information, and List of All Current EUAs. https://www.fda.gov/emergency-preparedness-and-response/mcm-legal-regulatory-and-policy-framework/emergency-use-authorization.

[22] Yang Y., Yang M., Yuan J., Wang F., Wang Z., Li J., Zhang M., Xing L., Wei J., Peng L. (2020). Comparative Sensitivity of Different Respiratory Specimen Types for Molecular Diagnosis and Monitoring of SARS-CoV-2 Shedding. Innovation.

[23] Zhao J., Yuan Q., Wang H., Liu W., Liao X., Su Y., Wang X., Yuan J., Li T., Li J. (2020). Antibody Responses to SARS-CoV-2 in Patients With Novel Coronavirus Disease 2019. Clin. Infect. Dis..

[24] Arevalo-Rodriguez I., Buitrago-Garcia D., Simancas-Racines D., Zambrano-Achig P., Del Campo R., Ciapponi A., Sued O., Martinez-García L., Rutjes A.W., Low N. (2020). False-negative results of initial RT-PCR assays for COVID-19: A systematic review. PLoS ONE.

[25] Loeffelholz M.J., Alland D., Butler-Wu S.M., Pandey U., Perno C.F., Nava A., Carroll K.C., Mostafa H., Davies E., McEwan A. (2020). Multicenter Evaluation of the Cepheid Xpert Xpress SARS-CoV-2 Test. J. Clin. Microbiol..

[26] Lieberman J.A., Pepper G., Naccache S.N., Huang M.L., Jerome K.R., Greninger A.L. (2020). Comparison of Commercially Available and Laboratory-Developed Assays for In Vitro Detection of SARS-CoV-2 in Clinical Laboratories. J. Clin. Microbiol..

[27] Woelfel R., Corman V.M., Guggemos W., Seilmaier M., Zange S., Mueller M.A., Niemeyer D., Kelly T.C.J., Vollmar P., Rothe C. (2020). Clinical presentation, and virological assessment of hospitalized cases of coronavirus disease 2019 in a travel-associated transmission cluster. medRxiv.

[28] Wang W., Xu Y., Gao R., Lu R., Han K., Wu G., Tan W. (2020). Detection of SARS-CoV-2 in Different Types of Clinical Specimens. JAMA.

[29] Bwire G.M., Majigo M.V., Njiro B.J., Mawazo A. (2021). Detection profile of SARS-CoV-2 using RT-PCR in different types of clinical specimens: A systematic review and meta-analysis. J. Med. Virol..

[30] Wyllie A.L., Fournier J., Casanovas-Massana A., Campbell M., Warren J.L., Geng B., Muenker M.C., Moore A.J., Vogels C.B.F., Petrone M.E. (2020). Saliva or nasopharyngeal swab specimens for detection of SARS-CoV-2. N. Engl. J. Med..

[31] Rao M., Rashid F.A., Sabri F.S.A.H., Jamil N.N., Zain R., Hashim R., Amran F., Kok H.T., Samad M.A.A., Ahmad N. (2021). Comparing Nasopharyngeal Swab and Early Morning Saliva for the Identification of Severe Acute Respiratory Syndrome Coronavirus 2 (SARS-CoV-2). Clin. Infect. Dis..

[32] Pasomsub E., Watcharananan S.P., Boonyawat K., Janchompoo P., Wongtabtim G., Suksuwan W., Sungkanuparph S., Phuphuakrat A. (2021). Saliva sample as a non-invasive specimen for the diagnosis of coronavirus disease 2019: A cross-sectional study. Clin. Microbiol. Infect..

[33] To K.K., Tsang O.T., Yip C.C., Chan K.H., Wu T.C., Chan J.M., Leung W.S., Chik T.S., Choi C.Y., Kandamby D.H. (2020). Consistent Detection of 2019 Novel Coronavirus in Saliva. Clin. Infect. Dis..

[34] Azzi L., Carcano G., Gianfagna F., Grossi P., Gasperina D.D., Genoni A., Fasano M., Sessa F., Tettamanti L., Carinci F. (2020). Saliva is a reliable tool to detect SARS-CoV-2. J. Infect..

[35] Becker D., Sandoval E., Amin A., De Hoff P., Diets A., Leonetti N., Lim Y.W., Elliott C., Laurent L., Grzymski J. (2020). Saliva is less sensitive than nasopharyngeal swabs for COVID-19 detection in the community setting. MedRxiv.

[36] Butler-Laporte G., Lawandi A., Schiller I., Yao M., Dendukuri N., McDonald E.G., Lee T.C. (2021). Comparison of Saliva and Nasopharyngeal Swab Nucleic Acid Amplification Testing for Detection of SARS-CoV-2: A Systematic Review and Meta-analysis. JAMA Intern. Med..

[37] Nagura-Ikeda M., Imai K., Tabata S., Miyoshi K., Murahara N., Mizuno T., Horiuchi M., Kato K., Imoto Y., Iwata M. (2020). Clinical Evaluation of Self-Collected Saliva by Quantitative Reverse Transcription-PCR (RT-qPCR), Direct RT-qPCR, Reverse Transcription-Loop-Mediated Isothermal Amplification, and a Rapid Antigen Test To Diagnose COVID-19. J. Clin. Microbiol..

[38] Yi H. (2020). 2019 Novel Coronavirus Is Undergoing Active Recombination. Clin. Infect. Dis..

[39] Phan T. (2020). Genetic diversity and evolution of SARS-CoV-2. Infect. Genet Evol..

[40] Pachetti M., Marini B., Benedetti F., Giudici F., Mauro E., Storici P., Masciovecchio C., Angeletti S., Ciccozzi M., Gallo R.C. (2020). Emerging SARS-CoV-2 mutation hot spots include a novel RNA-dependent-RNA polymerase variant. J. Transl. Med..

[41] Osório N.S., Correia-Neves M. (2020). Implication of SARS-CoV-2 evolution in the sensitivity of RT-qPCR diagnostic assays. Lancet Infect. Dis..

[42] Shen Z., Xiao Y., Kang L., Ma W., Shi L., Zhang L., Zhou Z., Yang J., Zhong J., Yang D. (2020). Genomic Diversity of Severe Acute Respiratory Syndrome-Coronavirus 2 in Patients With Coronavirus Disease 2019. Clin. Infect. Dis..

[43] Jiang F., Deng L., Zhang L., Cai Y., Cheung C.W., Xia Z. (2020). Review of the Clinical Characteristics of Coronavirus Disease 2019 (COVID-19). J. Gen. Intern. Med..

[44] Krishnan A., Hamilton J.P., Alqahtani S.A., A Woreta T. (2021). A narrative review of coronavirus disease 2019 (COVID-19): Clinical, epidemiological characteristics, and systemic manifestations. Intern. Emerg. Med..

[45] Afzal A. (2020). Molecular diagnostic technologies for COVID-19: Limitations and challenges. J. Adv. Res..

[46] Kim J.Y., Ko J.H., Kim Y., Kim Y.J., Kim J.M., Chung Y.S., Kim H.M., Han M.G., Kim S.Y., Chin B.S. (2020). Viral Load Kinetics of SARS-CoV-2 Infection in First Two Patients in Korea. J. Korean Med. Sci..

[47] Sethuraman N., Jeremiah S.S., Ryo A. (2020). Interpreting Diagnostic Tests for SARS-CoV-2. JAMA.

[48] Kucirka L.M., Lauer S.A., Laeyendecker O., Boon D., Lessler J. (2020). Variation in False-Negative Rate of Reverse Transcriptase Polymerase Chain Reaction-Based SARS-CoV-2 Tests by Time Since Exposure. Ann. Intern. Med..

[49] Poon L.L., Chan K.H., Wong O.K., Yam W.C., Yuen K.Y., Guan Y., Lo Y.M., Peiris J.S. (2003). Early diagnosis of SARS coronavirus infection by real time RT-PCR. J. Clin. Virol..

[50] de Sousa R., Reusken C., Koopmans M. (2014). MERS coronavirus: Data gaps for laboratory preparedness. J. Clin. Virol..

[51] Kobayashi T., Jung S.M., Linton N.M., Kinoshita R., Hayashi K., Miyama T., Anzai A., Yang Y., Yuan B., Akhmetzhanov A.R. (2020). Communicating the Risk of Death from Novel Coronavirus Disease (COVID-19). J. Clin. Med..

[52] Ferretti L., Wymant C., Kendall M., Zhao L., Nurtay A., Abeler-Dörner L., Parker M., Bonsall D., Fraser C. (2020). Quantifying SARS-CoV-2 transmission suggests epidemic control with digital contact tracing. Science.

[53] Cevik M., Tate M., Lloyd O., Maraolo A.E., Schafers J., Ho A. (2021). SARS-CoV-2, SARS-CoV, and MERS-CoV viral load dynamics, duration of viral shedding, and infectiousness: A systematic review and meta-analysis. Lancet Microbe.

[54] Inglis T.J. (2007). Principia aetiologica: Taking causality beyond Koch’s postulates. J. Med Microbiol..

[55] Sia S.F., Yan L.M., Chin A.W.H., Fung K., Choy K.T., Wong A.Y.L., Kaewpreedee P., Perera R.A.P.M., Poon L.L.M., Nicholls J.M. (2020). Pathogenesis and transmission of SARS-CoV-2 in golden hamsters. Nature.

[56] Wölfel R., Corman V.M., Guggemos W., Seilmaier M., Zange S., Müller M.A., Niemeyer D., Jones T.C., Vollmar P., Rothe C. (2020). Virological assessment of hospitalized patients with COVID-2019. Nature.

[57] Lambert-Niclot S., Cuffel A., Le Pape S., Vauloup-Fellous C., Morand-Joubert L., Roque-Afonso A.M., Le Goff J., Delaugerre C. (2020). Evaluation of a Rapid Diagnostic Assay for Detection of SARS-CoV-2 Antigen in Nasopharyngeal Swabs. J. Clin. Microbiol..

[58] Manabe Y.C., Sharfstein J.S., Armstrong K. (2020). The Need for More and Better Testing for COVID-19. JAMA.

[59] Pekosz A., Parvu V., Li M., Andrews J.C., Manabe Y.C., Kodsi S., Gary D.S., Roger-Dalbert C., Leitch J., Cooper C.K. (2021). Antigen-Based Testing but Not Real-Time Polymerase Chain Reaction Correlates With Severe Acute Respiratory Syndrome Coronavirus 2 Viral Culture. Clin. Infect. Dis..

[60] Strong J.E., Feldmann H. (2017). The crux of Ebola diagnostics. J. Infect. Dis..

[61] Bullard J., Dust K., Funk D., Strong J.E., Alexander D., Garnett L., Boodman C., Bello A., Hedley A., Schiffman Z. (2020). Predicting Infectious Severe Acute Respiratory Syndrome Coronavirus 2 From Diagnostic Samples. Clin. Infect. Dis..

[62] Yu F., Yan L., Wang N., Yang S., Wang L., Tang Y., Gao G., Wang S., Ma C., Xie R. (2020). Quantitative Detection and Viral Load Analysis of SARS-CoV-2 in Infected Patients. Clin. Infect. Dis..

[63] Jaafar R., Aherfi S., Wurtz N., Grimaldier C., Van Hoang T., Colson P., Raoult D., La Scola B. (2021). Correlation Between 3790 Quantitative Polymerase Chain Reaction-Positives Samples and Positive Cell Cultures, Including 1941 Severe Acute Respiratory Syndrome Coronavirus 2 Isolates. Clin. Infect. Dis..

[64] Mina M.J., Parker R., Larremore D.B. (2020). Rethinking Covid-19 Test Sensitivity—A Strategy for Containment. N. Engl. J. Med..

[65] COVID-19 Investigation Team (2020). Clinical and virologic characteristics of the first 12 patients with coronavirus disease 2019 (COVID-19) in the United States. Nat. Med..

[66] Arons M.M., Hatfield K.M., Reddy S.C., Kimball A., James A., Jacobs J.R., Taylor J., Spicer K., Bardossy A.C., Oakley L.P. (2020). Presymptomatic SARS-CoV-2 Infections and Transmission in a Skilled Nursing Facility. N. Engl. J. Med..

[67] Zou L., Ruan F., Huang M., Liang L., Huang H., Hong Z., Yu J., Kang M., Song Y., Xia J. (2020). SARS-CoV-2 Viral Load in Upper Respiratory Specimens of Infected Patients. N. Engl. J. Med..

[68] Lednicky J.A. (2020). A practical and economic approach for assessing potential SARS-CoV-2 transmission risk in COVID-19 patients. Clin. Infect. Dis..

[69] Lu J., Peng J., Xiong Q., Liu Z., Lin H., Tan X., Kang M., Yuan R., Zeng L., Zhou P. (2020). Clinical, immunological, and virological characterization of COVID-19 patients that test re-positive for SARS-CoV-2 by RT-PCR. Medrxiv.

[70] Korea Centers for Disease Control and Prevention Findings from Investigation and Analysis of Re-Positive Cases. 19 May 2020. https://www.cdc.go.kr/board/board.es?mid=a30402000000&bid=0030&act=view&list_no=367267&nPage=1external.

[71] https://www.fda.gov/media/134922/download.

[72] La Scola B., Le Bideau M., Andreani J., Hoang V.T., Grimaldier C., Colson P., Gautret P., Raoult D. (2020). Viral RNA load as determined by cell culture as a management tool for discharge of SARS-CoV-2 patients from infectious disease wards. Eur. J. Clin. Microbiol. Infect. Dis..

[73] Singanayagam A., Patel M., Charlett A., Lopez Bernal J., Saliba V., Ellis J., Ladhani S., Zambon M., Gopal R. (2020). Duration of infectiousness and correlation with RT-PCR cycle threshold values in cases of COVID-19, England, January to May 2020. Eurosurveillance.

[74] van Kampen J.J.A., van de Vijver D.A.M.C., Fraaij P.L.A., Haagmans B.L., Lamers M.M., Okba N., van den Akker J.P.C., Endeman H., Gommers D.A.M.P.J., Cornelissen J.J. (2021). Duration and key determinants of infectious virus shedding in hospitalized patients with coronavirus disease-2019 (COVID-19). Nat. Commun..

[75] Hicks J., Klumpp-Thomas C., Kalish H., Shunmugavel A., Mehalko J., Denson J.P., Snead K.R., Drew M., Corbett K.S., Graham B.S. (2021). Serologic Cross-Reactivity of SARS-CoV-2 with Endemic and Seasonal Betacoronaviruses. J. Clin. Immunol..

[76] Ma Z., Li P., Ji Y., Ikram A., Pan Q. (2020). Cross-reactivity towards SARS-CoV-2: The potential role of low-pathogenic human coronaviruses. Lancet Microbe.

[77] Your Coronavirus Test Is Positive. Maybe It Shouldn’t Be. The New York Times, August 2020. https://www.nytimes.com/2020/08/29/health/coronavirus-testing.html.

[78] He X., Lau E.H.Y., Wu P., Deng X., Wang J., Hao X., Lau Y.C., Wong J.Y., Guan Y., Tan X. (2020). Temporal dynamics in viral shedding and transmissibility of COVID-19. Nat. Med..

[79] Borczuk A.C., Salvatore S.P., Seshan S.V., Patel S.S., Bussel J.B., Mostyka M., Elsoukkary S., He B., Del Vecchio C., Fortarezza F. (2020). COVID-19 pulmonary pathology: A multi-institutional autopsy cohort from Italy and New York City. Mod. Pathol..

[80] Henderson D.K., Weber D.J., Babcock H., Hayden M.K., Malani A., Wright S.B., Murthy A.R., Guzman-Cottrill J., Haessler S., Rock C. (2021). The perplexing problem of persistently PCR-positive personnel. Infect. Control Hosp. Epidemiol..

[81] Pan Y., Long L., Zhang D., Yuan T., Cui S., Yang P., Wang Q., Ren S. (2020). Potential False-Negative Nucleic Acid Testing Results for Severe Acute Respiratory Syndrome Coronavirus 2 from Thermal Inactivation of Samples with Low Viral Loads. Clin. Chem..

[82] Guo L., Sun X., Wang X., Liang C., Jiang H., Gao Q., Dai M., Qu B., Fang S., Mao Y. (2020). SARS-CoV-2 detection with CRISPR diagnostics. Cell Discov..

[83] Huang Z., Tian D., Liu Y., Lin Z., Lyon C.J., Lai W., Fusco D., Drouin A., Yin X., Hu T. (2020). Ultra-sensitive and high-throughput CRISPR-p owered COVID-19 diagnosis. Biosens. Bioelectron..

[84] Tom M.R., Mina M.J. (2020). To Interpret the SARS-CoV-2 Test, Consider the Cycle Threshold Value. Clin. Infect. Dis..

[85] Struyf T., Deeks J.J., Dinnes J., Takwoingi Y., Davenport C., Leeflang M.M., Spijker R., Hooft L., Emperador D., Dittrich S. (2020). Cochrane COVID-19 Diagnostic Test Accuracy Group. Signs and symptoms to determine if a patient presenting in primary care or hospital outpatient settings has COVID-19 disease. Cochrane Database Syst. Rev..

[86] https://www.cebm.net/covid-19/when-is-covid-covid/.

[87] Covid, Lombardia errore nel calcolo dell’Rt: Migliaia di guariti sono stati conteggiati come ancora positivi (“Covid, Lombardy, Error in the Calculation of the RT: Thousands of Recovered Have Been Counted as Still Positive”). https://www.corriere.it/cronache/21_gennaio_23/lombardia-errore-dati-migliaia-guariti-sono-stati-conteggiati-come-ancora-positivi-a94fa550-5d3f-11eb-ae12-b118d8ea2872.shtml.

[88] Shuren J., Stenzel T. (2020). Covid-19 Molecular Diagnostic Testing—Lessons Learned. N. Engl. J. Med..

[89] Watson J., Whiting P.F., Brush J.E. (2020). Interpreting a covid-19 test result. BMJ.

[90] European Centre for Disease Prevention and Control Transmission of COVID-19. 30 June 2020. https://www.ecdc.europa.eu/en/covid-19/latestevidence/transmission.

[91] Ioannidis J.P.A. (2020). Global perspective of COVID-19 epidemiology for a full-cycle pandemic. Eur. J. Clin. Investig..

[92] Ioannidis J.P.A., Cripps S., Tanner M.A. (2020). Forecasting for COVID-19 has failed. Int. J. Forecast.

[93] Bizzarri M., Di Traglia M., Giuliani A., Vestri A., Fedeli V., Prestininzi A. (2020). New statistical RI index allow to better track the dynamics of COVID-19 outbreak in Italy. Sci. Rep..

[94] Chu D.K., Akl E.A., Duda S., Solo K., Yaacoub S., Schünemann H.J., COVID-19 Systematic Urgent Review Group Effort (SURGE) study authors (2020). Physical distancing, face masks, and eye protection to prevent person-to-person transmission of SARS-CoV-2 and COVID-19: A systematic review and meta-analysis. Lancet.

